# Supplementary material for: Effects of climate change on the movement of future landfalling Texas tropical cyclones
Source: Nat Commun. 2020 Jul 3;11:3319. doi: 10.1038/s41467-020-17130-7 (PMC7334231; doi:10.1038/s41467-020-17130-7)
Supplement: Supplementary file 1 — Supplementary Information [file 41467_2020_17130_MOESM1_ESM.pdf]

Supplamentary Information

Effects of climate change on the movement of future landfalling Texas tropical cyclones

Hassanzadeh et al.

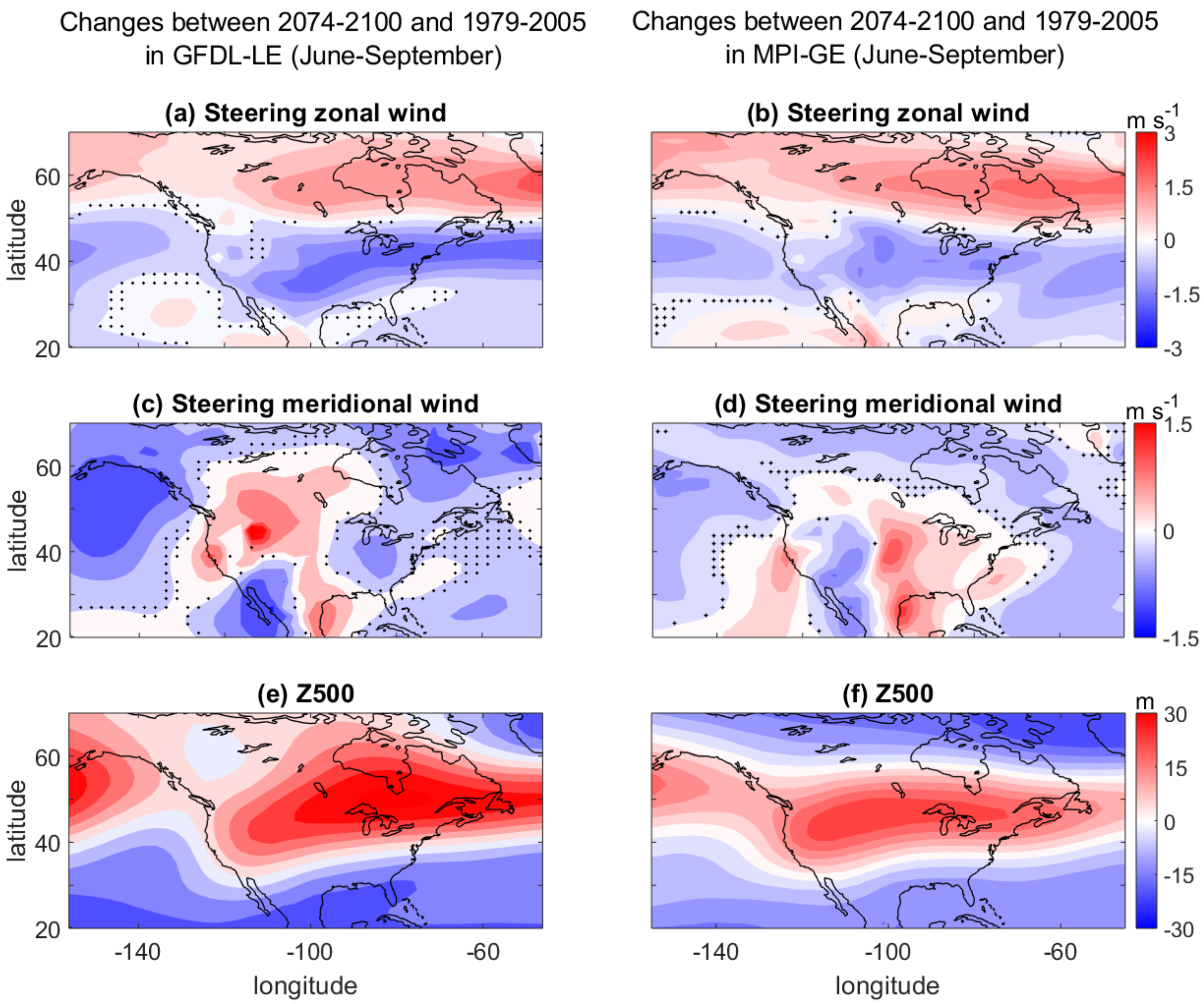

Supplementary Fig. 1: Changes in the large-scale circulation and steering winds under climate change. Changes are computed as June-September averages in the period of 2074-2100 minus those in the period of 1979-2005. (a), (c), and (e): Using 20 ensemble members of Geophysical Fluid Dynamical Laboratory Large Ensemble (GFDL-LE). A domain-averaged increase of 153.7 m is removed from geopotential height at 500 mb (Z500) for better illustration. (b), (d), and (f): Using 100 ensemble members of Max Planck Institute for Meteorology Grand Ensemble (MPI-GE). A domain-averaged increase of 86.2 m is removed from Z500 for better illustration. Stars show where the difference is not statistically significant, based on a two-tailed t test at 95% level. See Methods for further details.

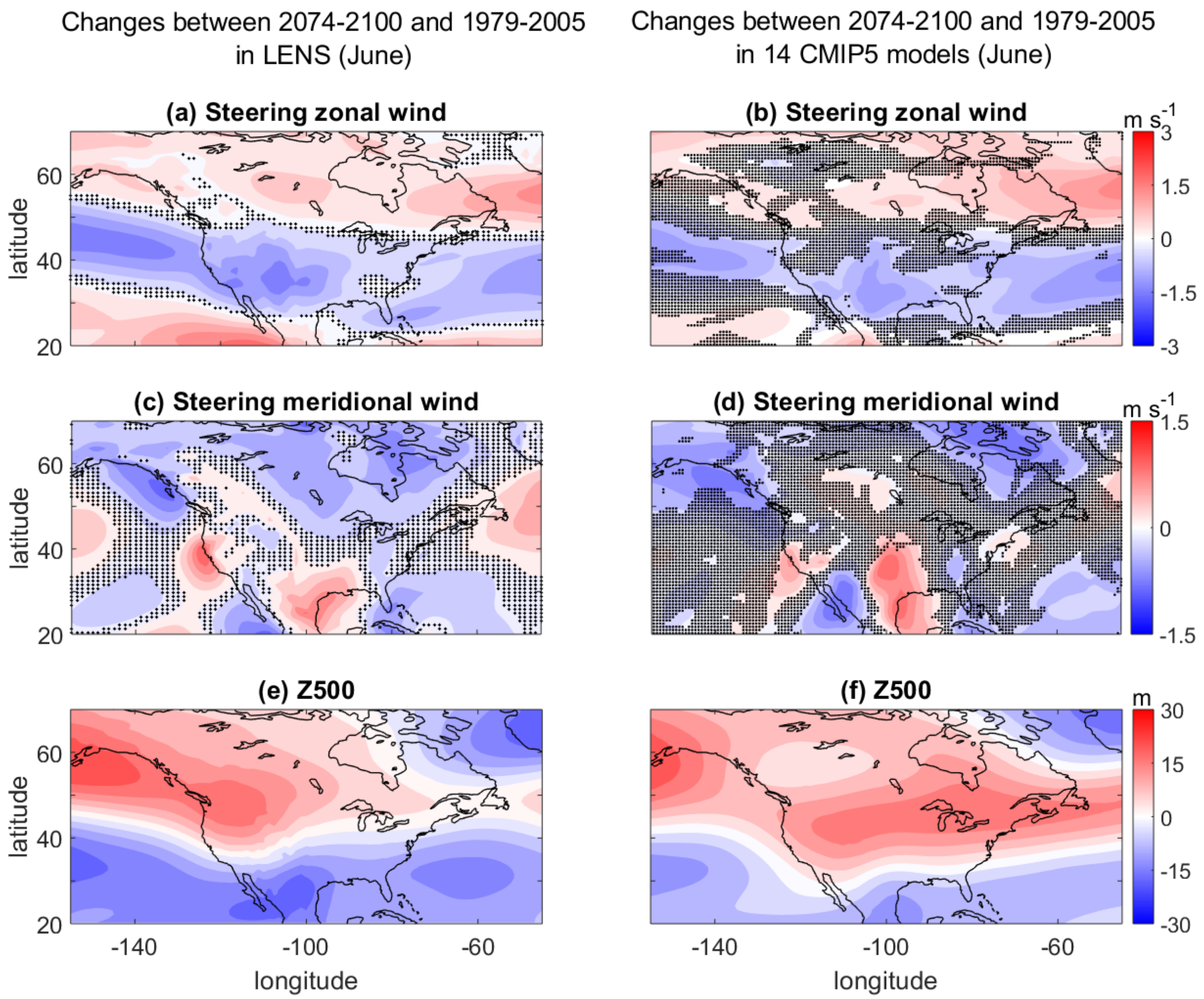

Supplementary Fig. 2: Changes in the large-scale circulation and steering winds under climate change in June. (a), (c), and (e): Using 40 ensemble members of National Center for Atmospheric Research's Large Ensemble Community Project (LENS). Stars show where the difference is not statistically significant, based on a two-tailed t test at 95% level. A domain-averaged increase of 108.7 m is removed from geopotential height at 500 mb (Z500). (b), (d), and (f): Using multi-model-mean from 14 Coupled Model Intercomparison Project 5 (CMIP5) models. Dots show where fewer than 10 models (out of 14) agree on the sign of the change. A domain-averaged increase of 121.1 m is removed from Z500. See Methods for further details.

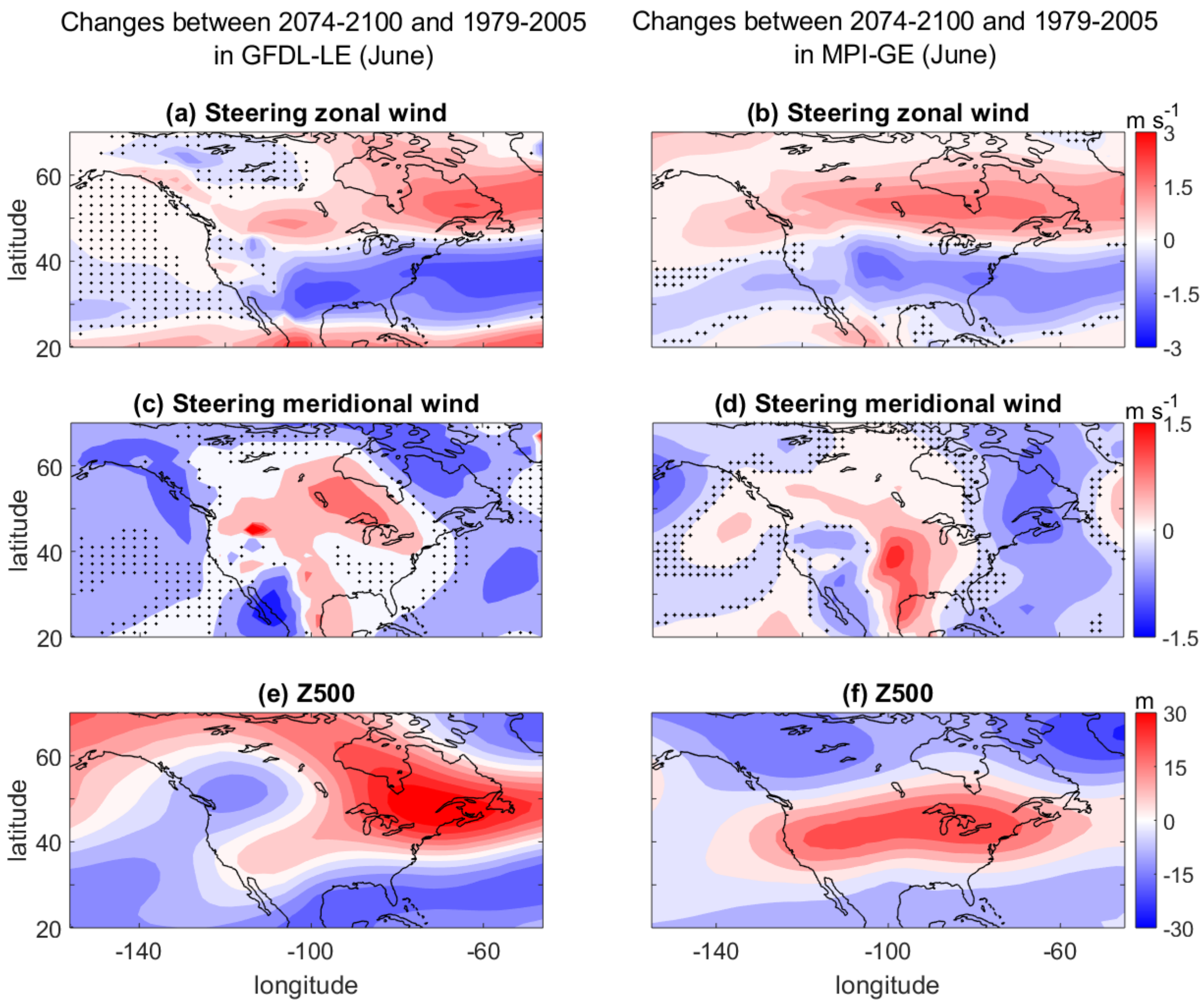

Supplementary Fig. 3: Changes in the large-scale circulation and steering winds under climate change in June. (a), (c), and (e): Using 20 ensemble members of Geophysical Fluid Dynamical Laboratory Large Ensemble (GFDL-LE). A domain-averaged increase of 143.1 m is removed from geopotential height at 500 mb (Z500). (b), (d), and (f): Using 100 ensemble members of Max Planck Institute for Meteorology Grand Ensemble (MPI-GE). A domain-averaged increase of 80.3 m is removed from Z500. Stars show where the difference is not statistically significant, based on a two-tailed t test at 95% level. See Methods for further details.

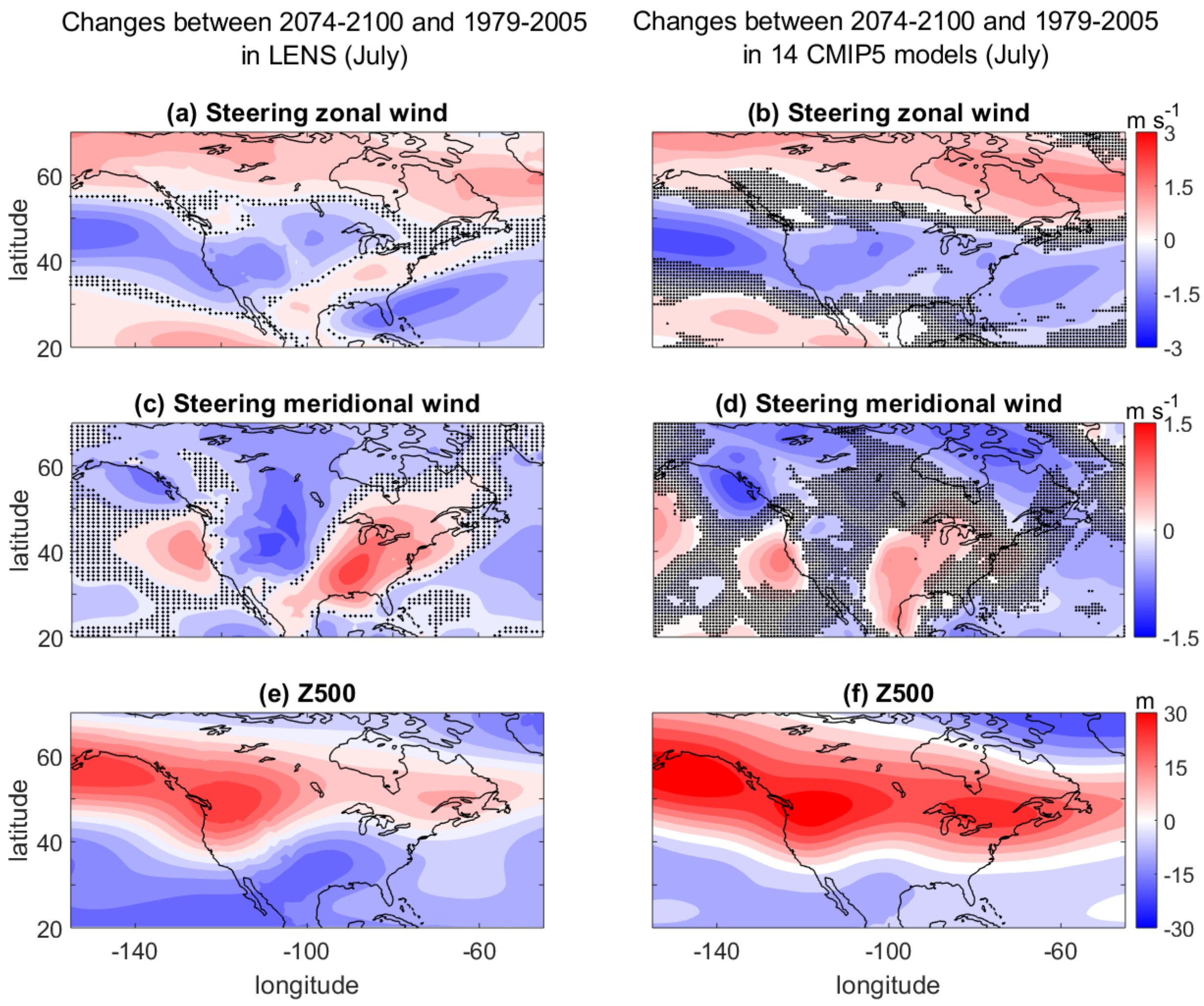

Supplementary Fig. 4: Changes in the large-scale circulation and steering winds under climate change in July. (a), (c), and (e): Using 40 ensemble members of National Center for Atmospheric Research's Large Ensemble Community Project (LENS). Stars show where the difference is not statistically significant, based on a two-tailed t test at 95% level. A domain-averaged increase of 114.4 m is removed from geopotential height at 500 mb (Z500). (b), (d), and (f): Using multi-model-mean from 14 Coupled Model Intercomparison Project 5 (CMIP5) models. Dots show where fewer than 10 models (out of 14) agree on the sign of the change. A domain-averaged increase of 123.2 m is removed from Z500. See Methods for further details.

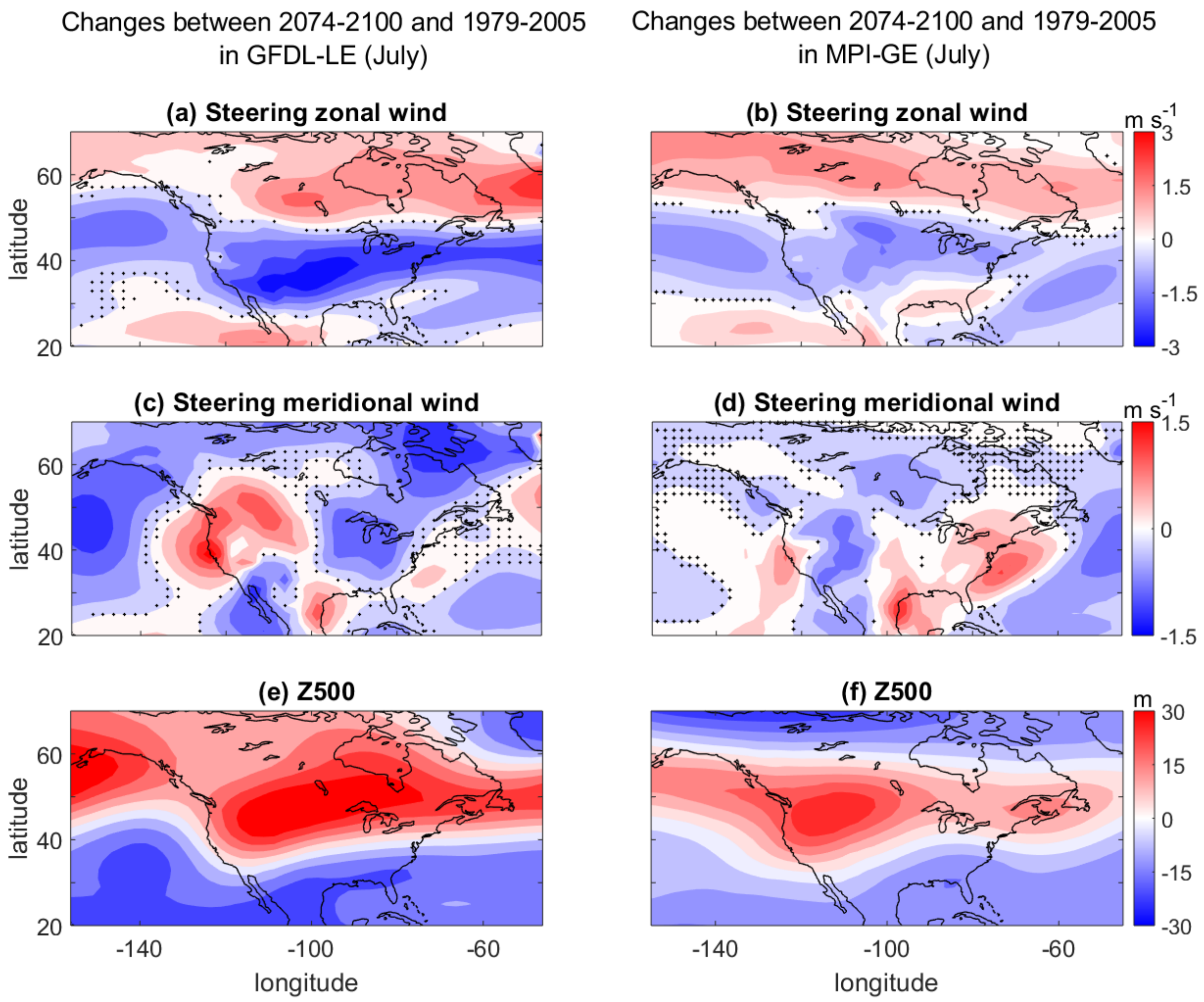

Supplementary Fig. 5: Changes in the large-scale circulation and steering winds under climate change in July. (a), (c), and (e): Using 20 ensemble members of Geophysical Fluid Dynamical Laboratory Large Ensemble (GFDL-LE). A domain-averaged increase of 156.8 m is removed from geopotential height at 500 mb (Z500). (b), (d), and (f): Using 100 ensemble members of Max Planck Institute for Meteorology Grand Ensemble (MPI-GE). A domain-averaged increase of 86.7 m is removed from Z500. Stars show where the difference is not statistically significant, based on a two-tailed t test at 95% level. See Methods for further details.

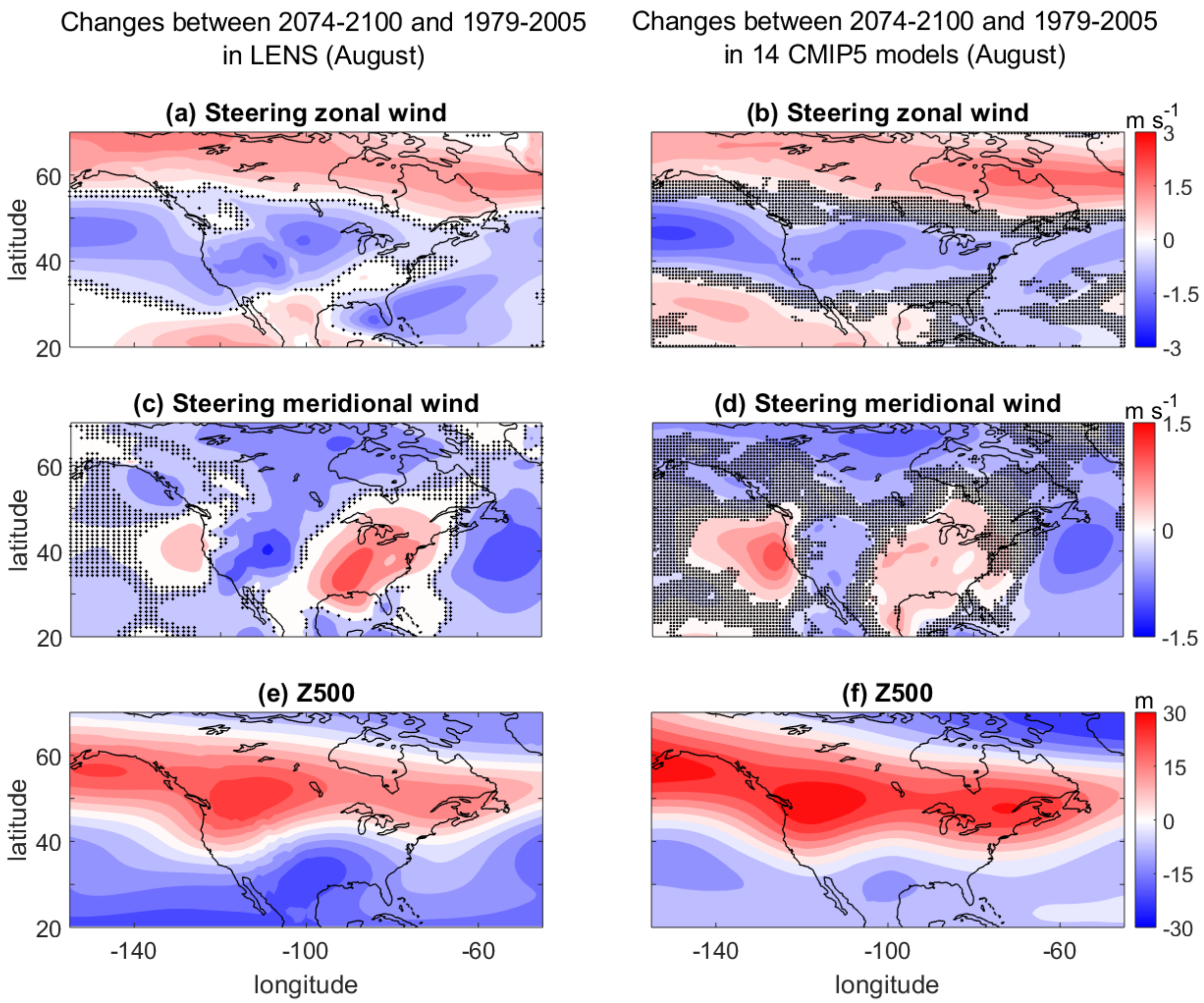

Supplementary Fig. 6: Changes in the large-scale circulation and steering winds under climate change in August. (a), (c), and (e): Using 40 ensemble members of National Center for Atmospheric Research's Large Ensemble Community Project (LENS). Stars show where the difference is not statistically significant, based on a two-tailed *t* test at 95% level. A domain-averaged increase of 114.0 m is removed from geopotential height at 500 mb (Z500) for better illustration. (b), (d), and (f): Using multi-model-mean from 14 Coupled Model Intercomparison Project 5 (CMIP5) models. Dots show where fewer than 10 models (out of 14) agree on the sign of the change. A domain-averaged increase of 124.1 m is removed from Z500. See Methods for further details.

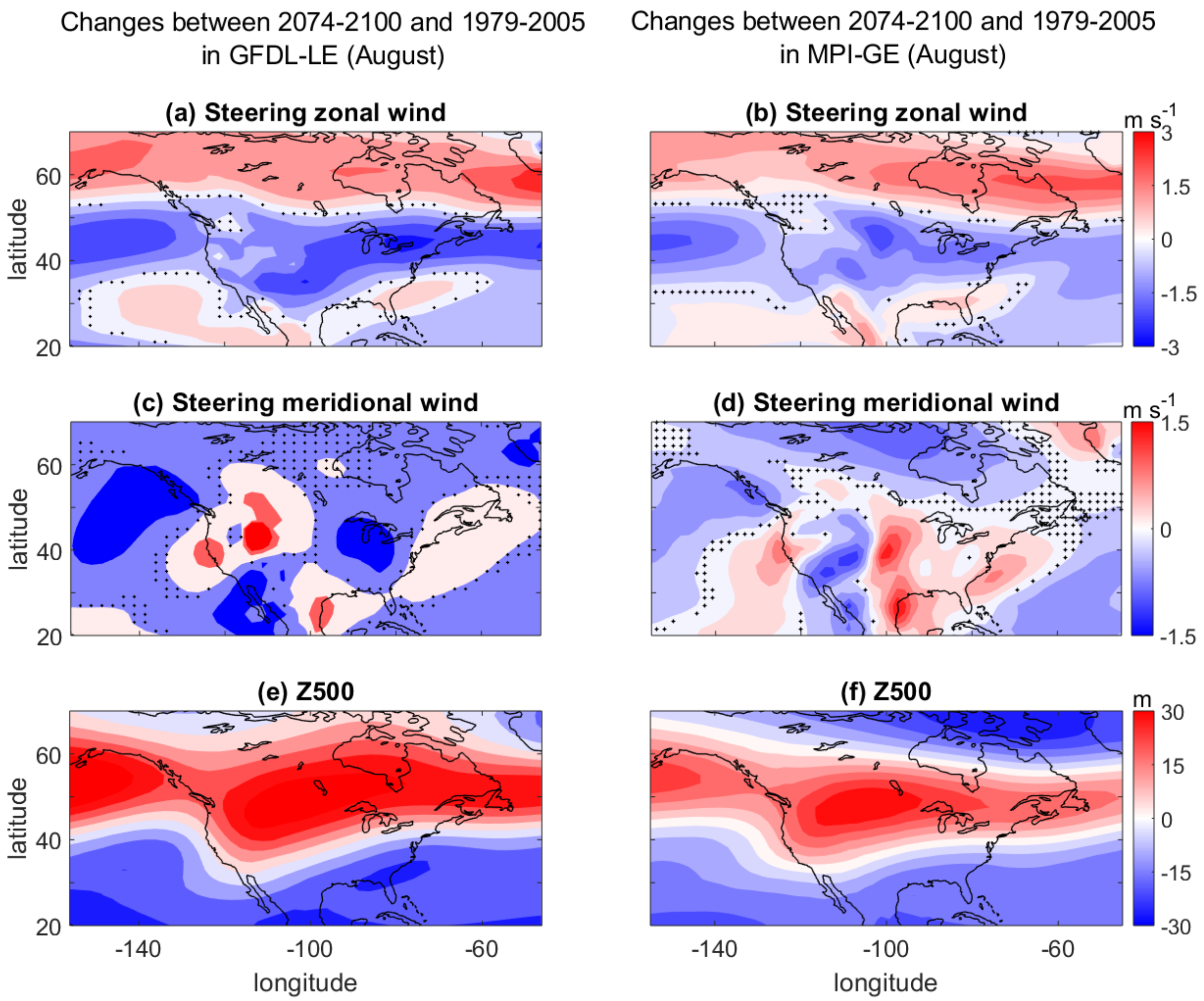

Supplementary Fig. 7: Changes in the large-scale circulation and steering winds under climate change in August. (a), (c), and (e): Using 20 ensemble members of Geophysical Fluid Dynamical Laboratory Large Ensemble (GFDL-LE). A domain-averaged increase of 162.3 m is removed from geopotential height at 500 mb (Z500). (b), (d), and (f): Using 100 ensemble members of Max Planck Institute for Meteorology Grand Ensemble (MPI-GE). A domain-averaged increase of 91.4 m is removed from Z500. Stars show where the difference is not statistically significant, based on a two-tailed *t* test at 95% level. See Methods for further details.

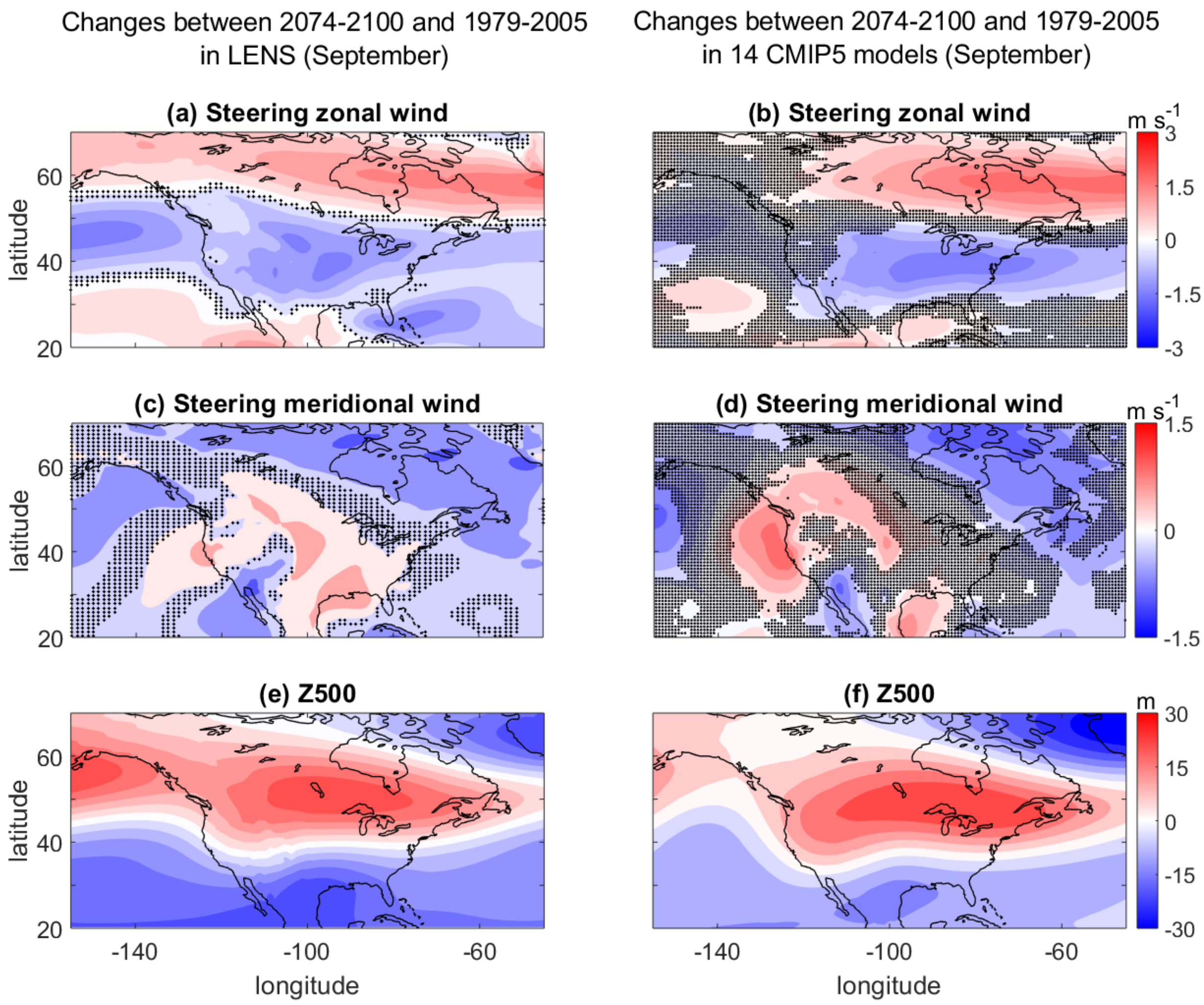

Supplementary Fig. 8: Changes in the large-scale circulation and steering winds under climate change in September. (a), (c), and (e): Using 40 ensemble members of National Center for Atmospheric Research's Large Ensemble Community Project (LENS). Stars show where the difference is not statistically significant, based on a two-tailed *t* test at 95% level. A domain-averaged increase of 113.3 m is removed from geopotential height at 500 mb (Z500). (b), (d), and (f): Using multi-model-mean from 14 Coupled Model Intercomparison Project 5 (CMIP5) models. Dots show where fewer than 10 models (out of 14) agree on the sign of the change. A domain-averaged increase of 122.3 m is removed from Z500. See Methods for further details.

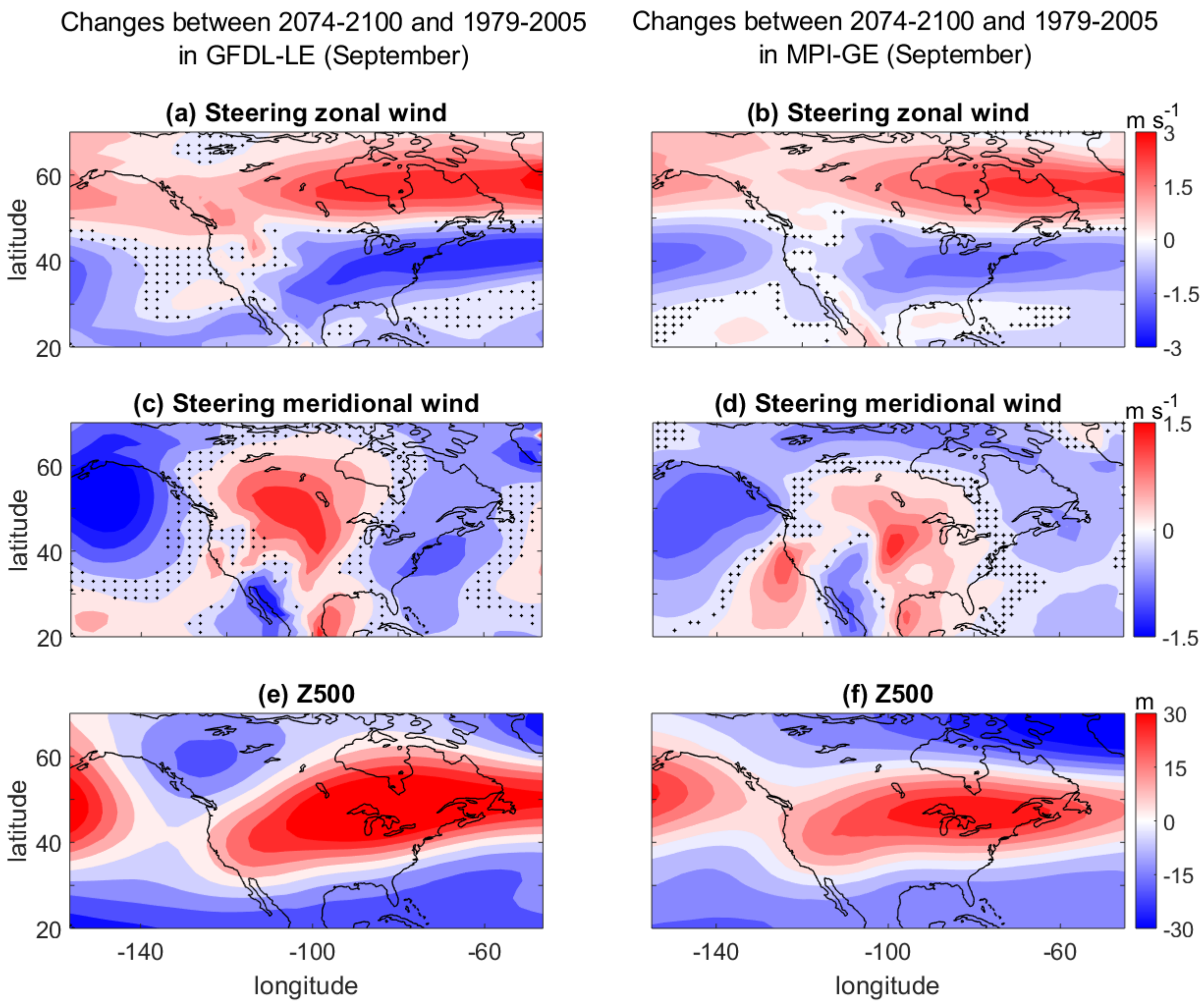

Supplementary Fig. 9: Changes in the large-scale circulation and steering winds under climate change in September. (a), (c), and (e): Using 20 ensemble members of Geophysical Fluid Dynamical Laboratory Large Ensemble (GFDL-LE). A domain-averaged increase of 152.6 m is removed from geopotential height at 500 mb (Z500). (b), (d), and (f): Using 100 ensemble members of Max Planck Institute for Meteorology Grand Ensemble (MPI-GE). A domain-averaged increase of 86.6 m is removed from Z500. Stars show where the difference is not statistically significant, based on a two-tailed t test at 95% level. See Methods for further details.

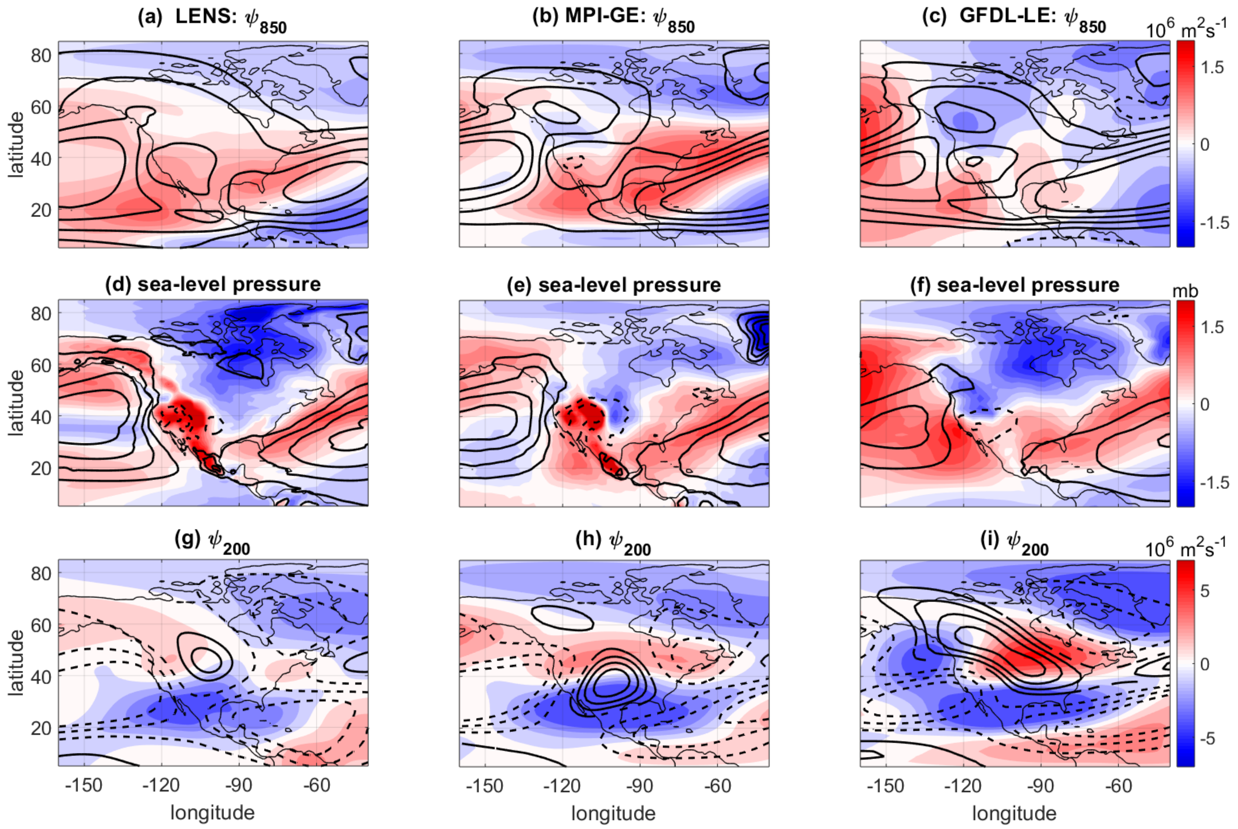

Supplementary Fig. 10: Changes in the stationary component of the large-scale circulation under climate change. Climatology (1979-2005, contours) and response (2074-2100 minus 1979-2005, shading) of the June-September-averaged stationary component of lower-level streamfunction ((a)-(c)), sea-level pressure ((d)-(f)), and upper-level streamfunction ((g)-(i)) for June-September from (a), (d), and (g): The 40 ensemble members of National Center for Atmospheric Research's Large Ensemble Community Project (LENS); (b), (e), and (h): The 100 ensemble members of Max Planck Institute for Meteorology Grand Ensemble (MPI-GE); (c), (f), and (i): The 20 ensemble members of Geophysical Fluid Dynamical Laboratory Large Ensemble (GFDL-LE). The contour intervals are 3×10^6 m^2 s^{-1} for streamfunctions and 2 mb for sea-level pressure. The stationary components are computed following Wills et al.[1].

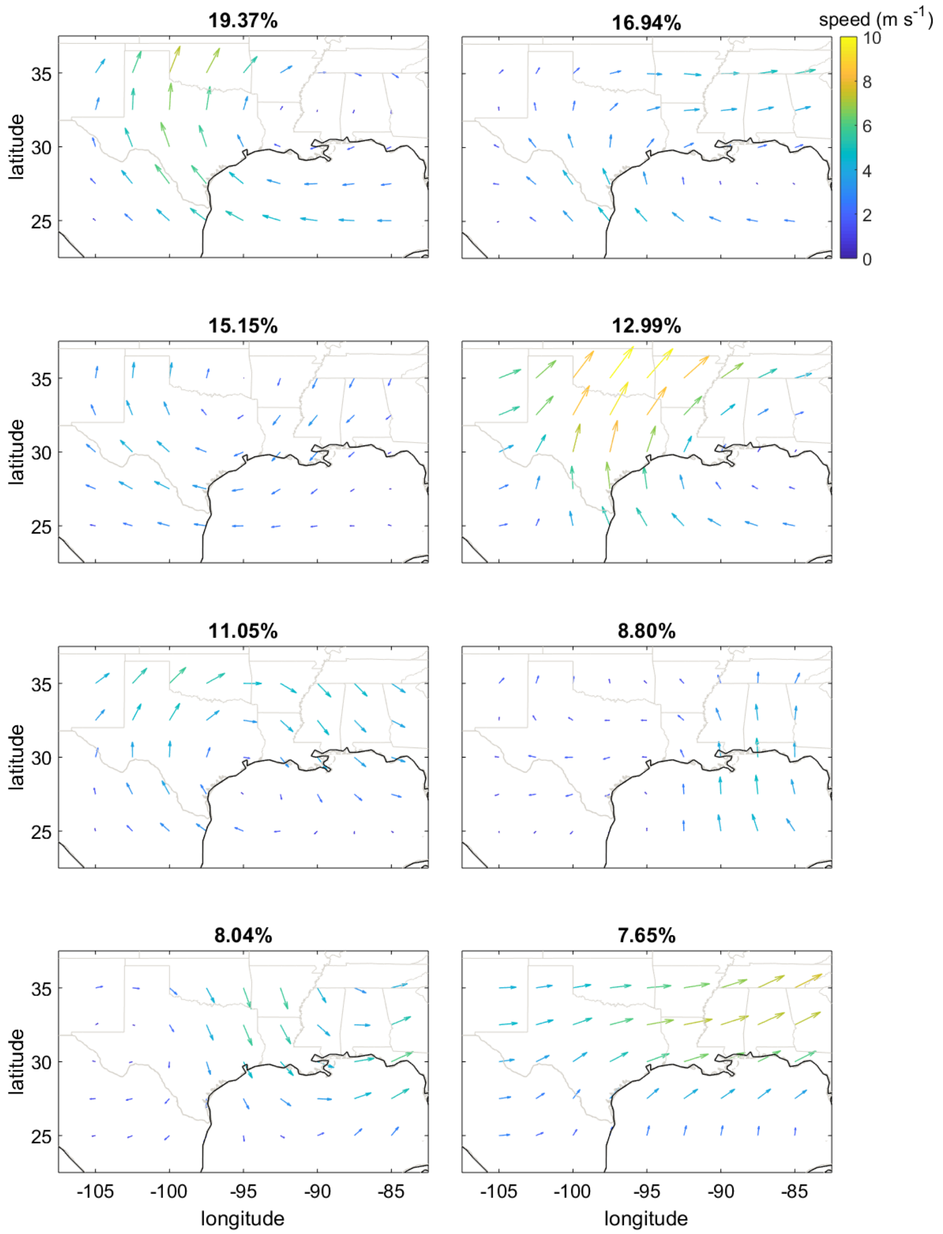

Supplementary Fig. 11: Clusters of daily steering wind patterns in NCEP-DOE reanalysis. Clusters are obtained from applying a self-organizing map (SOM) analysis to 1979-2005, June-September daily wind vectors from reanalysis data. Clusters are ordered based on their frequency. Note that the dimension of the SOM is 2×4, but presented as 4×2 for better illustration. See Methods for further details.

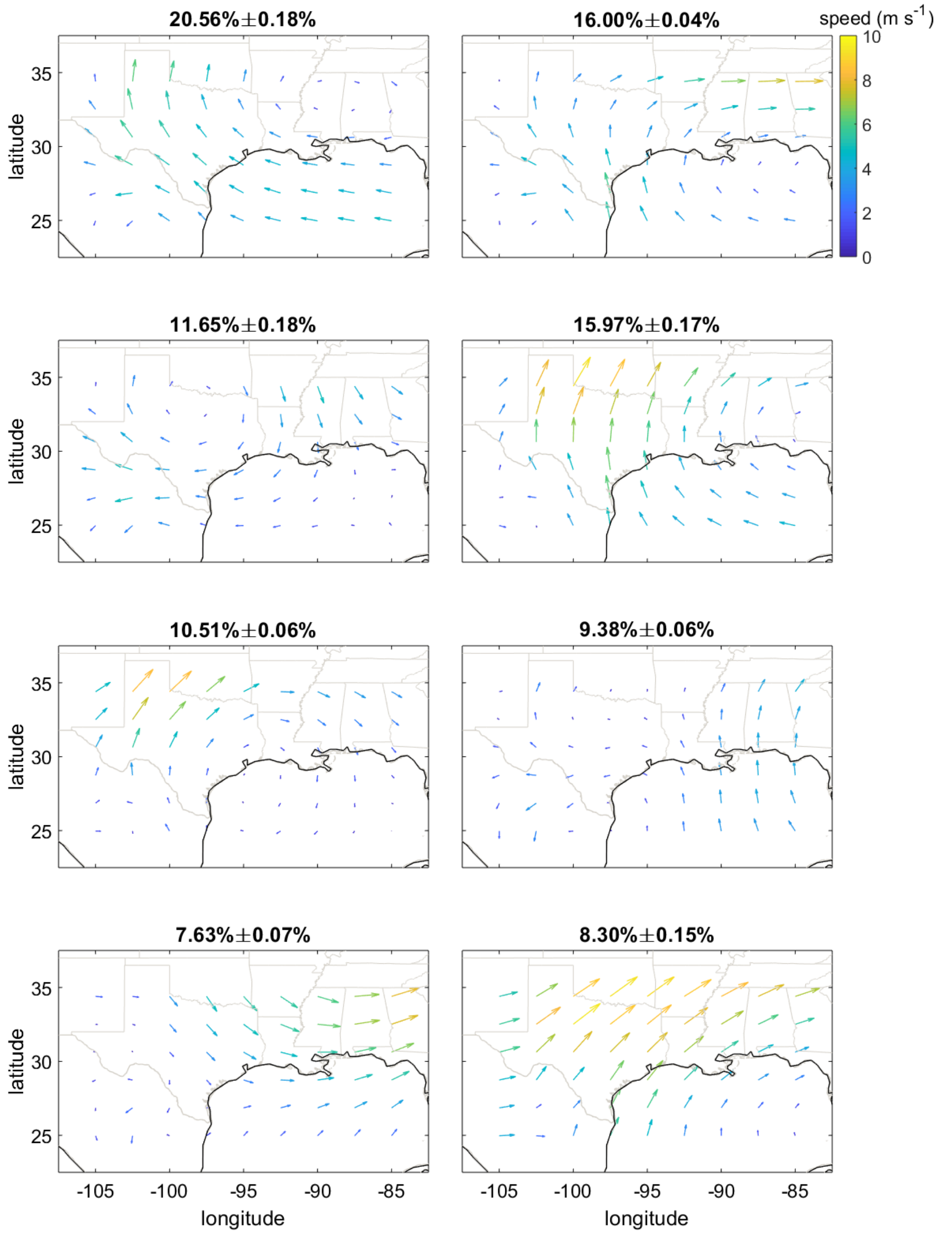

Supplamentart Fig. 12: Clusters of daily steering wind patterns in the Large Ensemble Community Project (LENS) dataset for the current climate. Clusters are obtained from applying a self-organizing map (SOM) analysis to 1979-2005, June-September daily wind vectors from National Center for Atmospheric Research's LENS data. Clusters are ordered based on their correspondence to the clusters of Supplamentary Fig. 11 (NCEP-DOE reanalysis). Comparing Supplamentary Figs. 11 and 12 shows that LENS reproduces the frequency and pattern of June-September steering wind regimes in reanalysis fairly well. To be quantitative, the pattern correlations (erorrs in frequency) between the corresponding panels of Suppalemntary Figs. 11 and 12, counting from left to right and then top to bottom, are 0.93 (6.1%), 0.94 (5.5%), 0.67 (23.1%), 0.91 (22.9%), 0.87 (4.9%), 0.93 (6.7%), 0.91 (5.1%), and 0.62 (8.5%). See Methods for further details.

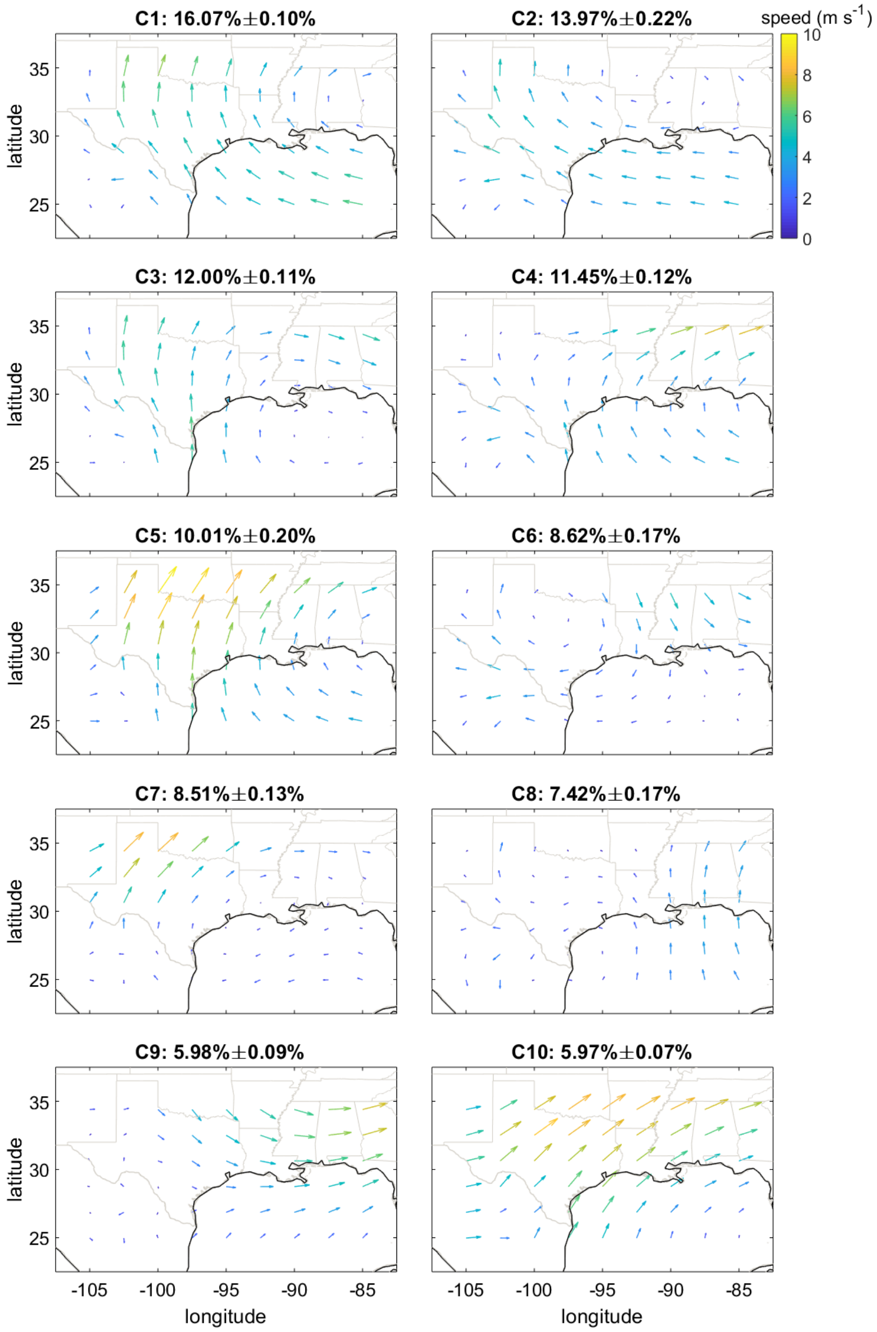

Supplametary Fig. 13: Clusters of daily steering wind patterns in Large Ensemble Community Project (LENS) dataset for the current and future climates combined. Daily June-September 1979-2005 and 2074-2100 steering wind patterns are clustered using self-organizing map (SOM) analysis. Clusters are ordered based on their frequency. Numbers show the frequency of each cluster $\bar{f_i}$ and its standard error. Clusters C1-C5, and C10 have strong northward steering winds over Texas while clusters C6 and C9 are the only ones with southward steering winds over Texas. Clusters C7 and C8 have weak steering winds over Texas. Note that in Supplamentary Figs. 13-16, the dimension of the SOM is 2×5, but presented as 5×2 for better illustration. See Methods for further details.

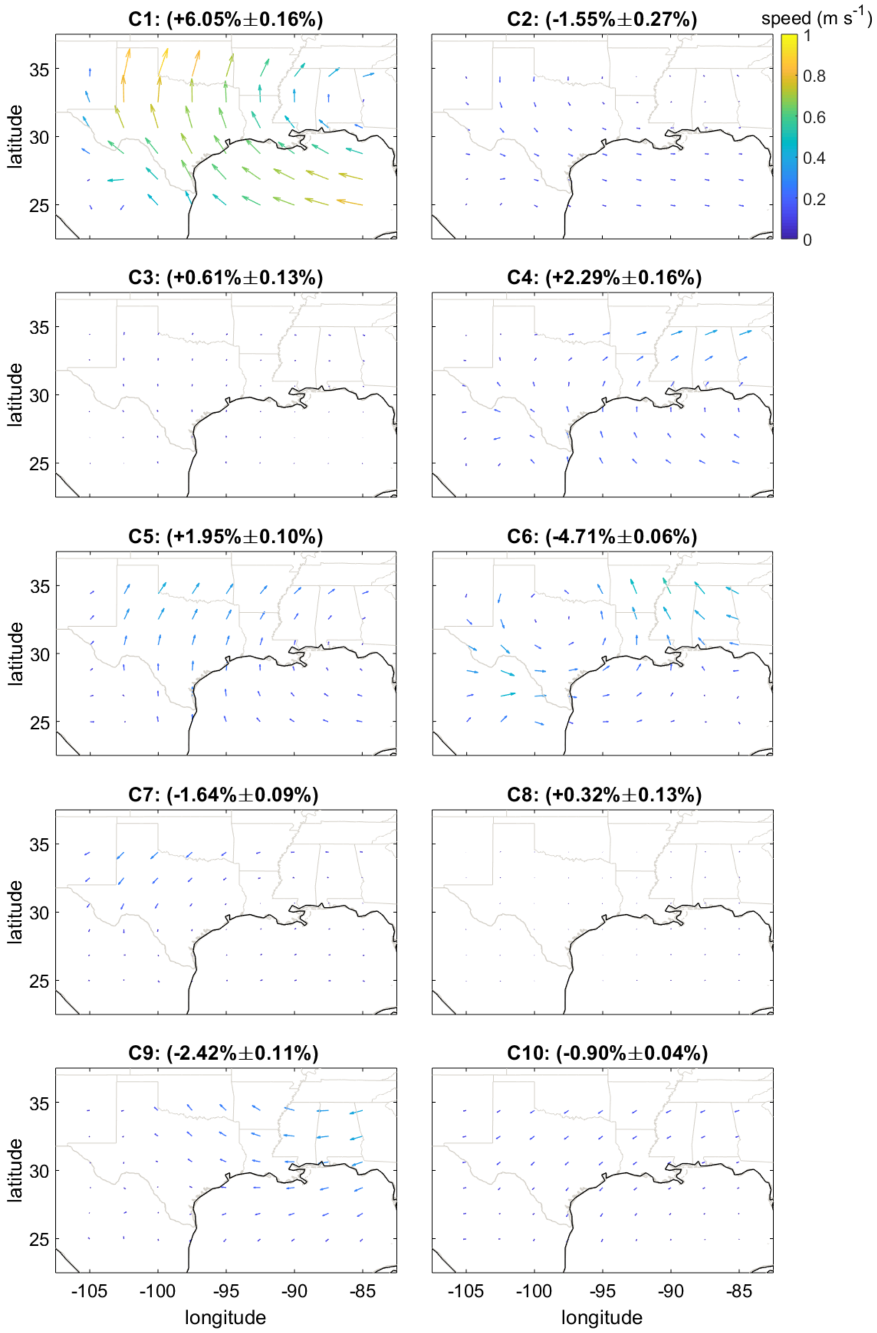

Supplementary Fig. 14: Same as Supplementary Fig. 13, but shows changes in each cluster between the future and current climates due to changes in frequency: $(\overline{P}_i \times \Delta f_i)$ in Eq. (4). Numbers show change in the frequency of each cluster (future minus current: $f_i^{\mathrm{F}} - f_i^{\mathrm{C}}$). The arrow size and colormap in Supplementary Figs. 14-16 are scaled such that their values are 1/10 of those in Supplementary Fig. 13. Increase (by ~7%) in the frequency of C1, C4, and C5 (which have northward steering winds over Texas, see Supplementary Fig. 12), and decrease (by ~7%) in the frequency of C6 and C9 (which both have southward steering winds over Texas, see Supplementary Fig. 12) in the future climate lead to an increase in northward steering winds over Texas. See Methods for further details.

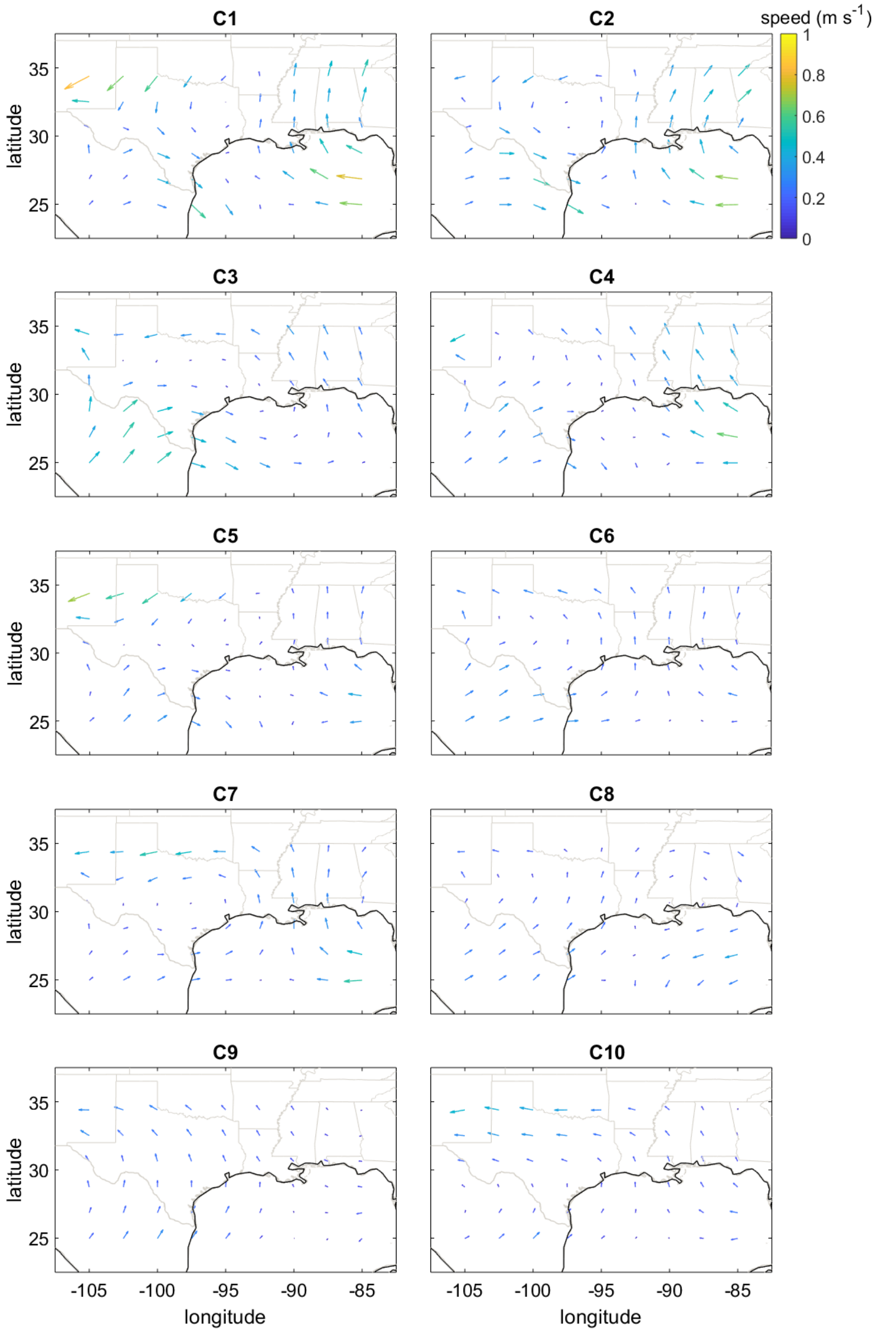

Supplementary Fig. 15: Same as Supplementary Fig. 13, but shows changes in each cluster between the future and current climates due to changes in pattern: $(\bar{f}_i \times \Delta P_i)$ in Eq. (5). In particular, note that the change in the patterns in C6 and C9 (which both have southward steering winds over Texas, see Supplementary Fig. 12) in the future climate lead to a decrease in southward steering winds over Texas. See Methods for further details.

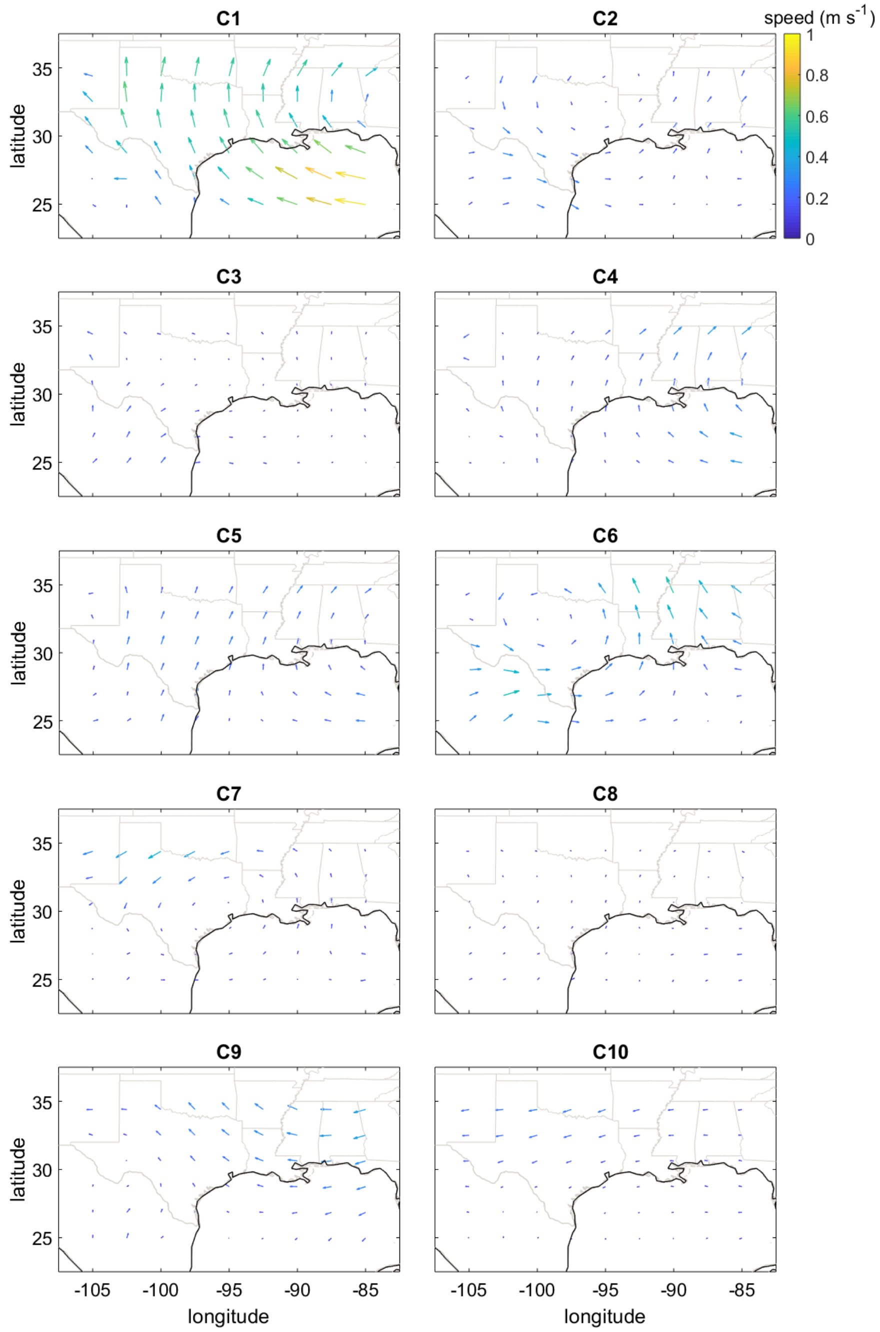

Supplementary Fig. 16: Same as Supplementary Fig. 13, but shows changes in each cluster between the future and current climates due to changes in frequency and in pattern (Eq. (3)). Each panel here shows the sum of the corresponding panels in Supplementary Figs. 14 and 15, and demonstrates how each panel in Supplementary Fig. 13 changes between the future and current climates. In particular, note that clusters C1, C4, C5 (which have northwards steering winds over Texas, see Supplementary Fig. 13) will have stronger northward steering winds, and clusters C6 and C9 (which have southward steering winds over Texas, see Supplementary Fig. 13) will have weaker southward steering winds over Texas. See Methods for further details.

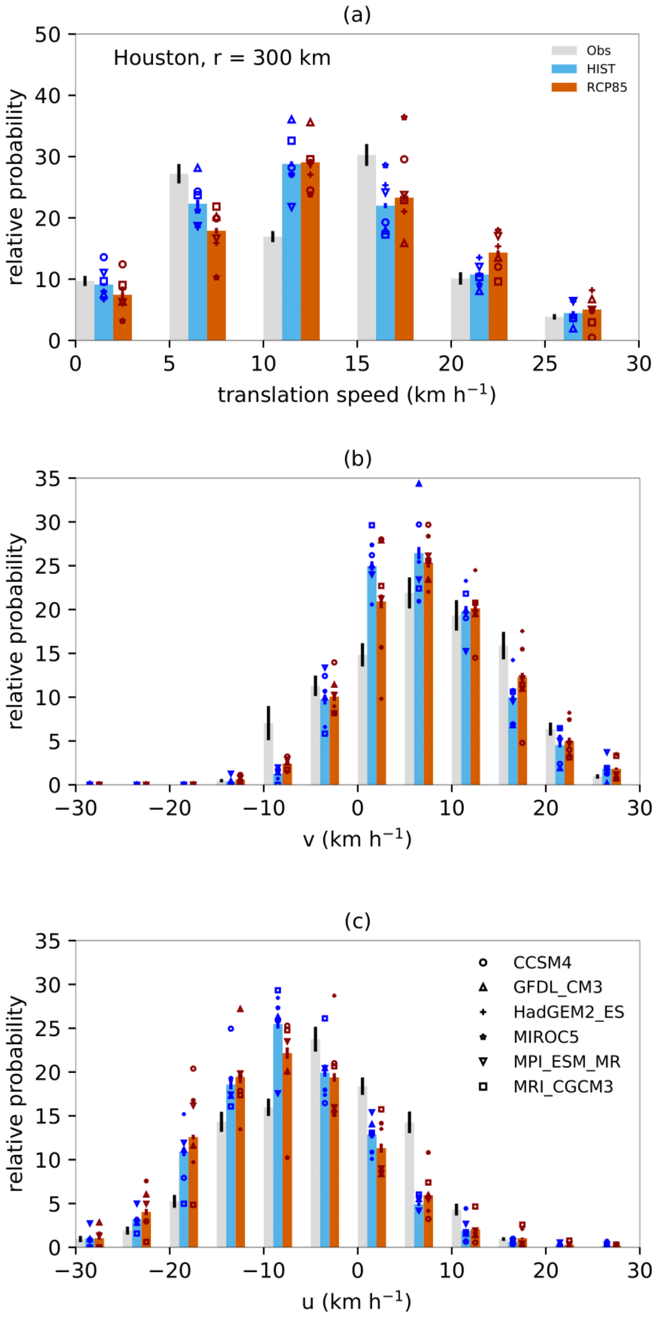

Supplementary Fig. 17: Same as Fig. 4 but with Gaussian bias correction. In (a), there is ~9.5% shift from the relative probability of slow-moving tropical cyclones (TCs, speed ≤ 5 km h^{-1}) toward that of fast-moving TCs (speed ≥ 20 km h^{-1}) under RCP8.5. In (b), there is ~7.8% shift from the relative probability of slow-moving TCs ($|v|$ ≤ 5 km h^{-1}) toward that of fast-moving TCs (northward speed v ≥ 15 km h^{-1}). In (c), there is ~4.4% shift from the relative probability of slow-moving TCs ($|u|$ ≤ 5 km h^{-1}) toward that of fast-moving TCs (westward speed u ≥ 15 km h^{-1}). With quantile-matching bias correction (not shown), these numbers are, 7.9%, 8.5%, and 4.8%, respectively, indicating a similar shift toward faster-moving TCs under RCP8.5. See Methods for further details.

Supplementary Table 1. The Coupled Model Intercomparison Project 5 (CMIP5) models used in this study. The first 14 models are used for computing the mean changes in large-scale circulation between the current and future climates (Fig. 2 and Supplementary Figs. 2, 4, 6, 8). The 6 models identified with shading are used in the CHAZ model for the downscaling experiments (Fig. 4 and Supplementary Fig. 17). Note that HadGEM2-ES is used in the downscaling.

Model	Resolution lon × lat	Number of vertical levels	Institute
ACCESS1.0	1.874° × 1.25°	38	Australian Community Climate and Earth-System Simulator, version 1.0[2]
ACCESS1.3	1.874° × 1.25°	38	Australian Community Climate and Earth-System Simulator, version 1.3[2]
BCC_CSM1.1(m)	1.25° × 1.25°	26	Beijing Climate Center, Climate System Model, version 1.1, moderate resolution[3]
BNU-ESM	2.8125° × 2.8125°	26	Beijing Normal University–Earth System Model[4]
CanESM2	2.8125° × 2.8125°	35	Second Generation Canadian Earth System Model[5]
CCSM4	1.25° × 0.937°	26	Community Climate System Model, version 4[6]
CMCC-CM	0.75° × 0.75°	31	Centro Euro-Mediterraneo sui Cambiamenti Climatici Climate Model[7]
CNRM-CM5	1.41° × 1.41°	31	Centre National de Recherches Météorologiques Coupled Global Climate Model, version 5[8]
GFDL-CM3	2.5° × 2°	48	Geophysical Fluid Dynamics Laboratory Climate Model, version 3[9]
GFDL-ESM2M	2.5° × 2°	48	Geophysical Fluid Dynamics Laboratory Earth System Model with MOM, version 4 component[10]
HadGEM2-CC<br>HadGEM2-ES	1.875° × 1.25°	60 (CC)<br>38 (ES)	UK Meteorological Office Hadley Centre Global Environment Model, version 2 Carbon Cycle (CC)[11]<br>Earth System (ES)[12]
IPSL-CM5A-MR	2.5° × 1.27°	39	L'Institut Pierre-Simon Laplace Coupled Model, version 5A, medium resolution[13]
MPI-ESM-MR	1.875° × 1.875°	95	Max Planck Institute Earth System Model, medium resolution[14]
MRI-CGCM3	1.125° × 1.125°	48	Meteorological Research Institute of Japan Climate General Circulation Model 3[15]
MIROC5	1.4° × 1.4°	40	University of Tokyo Center for Climate System Research Model for Interdisciplinary Research on Climate, version 5[16]

Supplementary References

1. Wills, R.C.J., White, R.H. & Levine, X.J. Northern hemisphere stationary waves in a changing climate. *Curr. Clim. Change Rep.* **5,** 372-389 (2019).
2. Collier, M. & Uhe, P. CMIP5 datasets from the ACCESS1. 0 and ACCESS1. 3 coupled climate models. *Technical Report, CAWCR Technical Report No. 059* (The Center for Australian Weather and Climate Research, 2012).
3. Wu, T. et al. The Beijing climate center atmospheric general circulation model: description and its performance for the present-day climate. *Clim. Dyn.* **34,** 123-147 (2010).
4. Ji, D. et al. Description and basic evaluation of Beijing normal university earth system model (BNU-ESM) version 1. *Geosci. Model Dev.* **7,** 2039-2064 (2014).
5. Arora, V.K. et al. Carbon emission limits required to satisfy future representative concentration pathways of greenhouse gases. *Geophys. Res. Lett.* **38,** L05805 (2011).
6. Gent, P.R. et al. The community climate system model version 4. *J. Clim.* **24,** 4973-4991 (2011).
7. Scoccimarro, E. et al. Effects of tropical cyclones on ocean heat transport in a high-resolution coupled general circulation model. *J. Clim.* **24,** 4368-4384 (2011).
8. Voldoire, A. et al. The CNRM-CM5. 1 global climate model: description and basic evaluation. *Clim. Dyn.* **40,** 2091-2121 (2013).
9. Donner, L.J. et al. The dynamical core, physical parameterizations, and basic simulation characteristics of the atmospheric component AM3 of the GFDL global coupled model CM3. *J. Clim.* **24,** 3484-3519 (2011).

10. Dunne, J.P. et al. GFDL's ESM2 global coupled climate–carbon earth system models. part I: physical formulation and baseline simulation characteristics. *J. Clim.* **25,** 6646-6665 (2012).

11. Bellouin, N. et al. The HadGEM2 family of met office unified model climate configurations. *Geosci. Model Dev.* **4,** 723-757 (2011).

12. Jones, C. et al. The HadGEM2-ES implementation of CMIP5 centennial simulations. *Geosci. Model Dev.* **4,** 543-570 (2011).

13. Dufresne, J.L. et al. Climate change projections using the IPSL-CM5 earth system model: from CMIP3 to CMIP5. *Clim. Dyn.* **40,** 2123-2165 (2013).

14. Zanchettin, D. et al. Bi-decadal variability excited in the coupled ocean–atmosphere system by strong tropical volcanic eruptions. *Clim. Dyn.* **39,** 419-444 (2012).

15. Yukimoto, S. et al. A new global climate model of the meteorological research institute: MRI-CGCM3—model description and basic performance. *J. Meteor. Soc. Japan* **90,** 23-64 (2012).

16. Watanabe, M. et al. Improved climate simulation by MIROC5: mean states, variability, and climate sensitivity. *J. Clim.* **23,** 6312-6335 (2010).
