# Supplementary material for: Effects of climate change on the movement of future landfalling Texas tropical cyclones
Source: Nat Commun. 2020 Jul 3;11:3319. doi: 10.1038/s41467-020-17130-7 (PMC7334231; doi:10.1038/s41467-020-17130-7)
Supplement: Supplementary file 3 — Description of Additional Supplementary Files [file 41467_2020_17130_MOESM3_ESM.pdf]

Description of Additional Supplementary Files

File Name: Supplementary Data 1

Description: Data used in Fig. 4. The file contains the speed and the zonal and meridional components of the translation speed from observation and from downscaling experiments under the historical and RCP8.5 forcings without any bias correction.

File Name: Supplementary Data 2

Description: Data used in Supplementary Fig. 17. The file contains the speed and the zonal and meridional components of the translation speed from observation and from downscaling experiments under the historical and RCP8.5 forcings with Gaussian bias correction.
